# Supplementary material for: Modeling glioblastoma heterogeneity as a dynamic network of cell states
Source: Mol Syst Biol. 2021 Sep 16;17(9):e10105. doi: 10.15252/msb.202010105 (PMC8444284; doi:10.15252/msb.202010105)
Supplement: Supplementary file 6 — Source Data for Figure 5 [file MSB-17-e10105-s004.zip › Figure5A_sourcedata/GSEA_3017/nosigdb_stateB.GseaPreranked.1622795021725/DOROSZKO_U3013MG_CLUSTER_2.html]

Details for gene set DOROSZKO\_U3013MG\_CLUSTER\_2[GSEA]

|  || Dataset | state43017 |
| Phenotype | NoPhenotypeAvailable |
| Upregulated in class | na\_pos |
| GeneSet | DOROSZKO\_U3013MG\_CLUSTER\_2 |
| Enrichment Score (ES) | 0.7079218 |
| Normalized Enrichment Score (NES) | 4.3681927 |
| Nominal p-value | 0.0 |
| FDR q-value | 0.0 |
| FWER p-Value | 0.0 |
Table: GSEA Results Summary

  

Fig 1: Enrichment plot: DOROSZKO\_U3013MG\_CLUSTER\_2      
 Profile of the Running ES Score & Positions of GeneSet Members on the Rank Ordered List

  

| PROBE | GENE SYMBOL | GENE\_TITLE | RANK IN GENE LIST | RANK METRIC SCORE | RUNNING ES | CORE ENRICHMENT || 1 | HIST1H4C |  |  | 0 | 1.373 | 0.0160 | Yes |
| 2 | TOP2A |  |  | 3 | 1.079 | 0.0253 | Yes |
| 3 | RRM2 |  |  | 7 | 0.875 | 0.0305 | Yes |
| 4 | FAM111A |  |  | 10 | 0.813 | 0.0366 | Yes |
| 5 | UBE2C |  |  | 11 | 0.802 | 0.0459 | Yes |
| 6 | TPX2 |  |  | 12 | 0.801 | 0.0553 | Yes |
| 7 | NUSAP1 |  |  | 13 | 0.801 | 0.0647 | Yes |
| 8 | HMGB2 |  |  | 14 | 0.786 | 0.0739 | Yes |
| 9 | NCAPG |  |  | 16 | 0.778 | 0.0813 | Yes |
| 10 | SPC25 |  |  | 17 | 0.763 | 0.0902 | Yes |
| 11 | PRC1 |  |  | 18 | 0.756 | 0.0990 | Yes |
| 12 | CDCA2 |  |  | 19 | 0.751 | 0.1078 | Yes |
| 13 | ESCO2 |  |  | 20 | 0.742 | 0.1164 | Yes |
| 14 | TACC3 |  |  | 21 | 0.741 | 0.1251 | Yes |
| 15 | GTSE1 |  |  | 22 | 0.732 | 0.1336 | Yes |
| 16 | HJURP |  |  | 23 | 0.731 | 0.1422 | Yes |
| 17 | KIF23 |  |  | 24 | 0.721 | 0.1506 | Yes |
| 18 | ASPM |  |  | 26 | 0.695 | 0.1570 | Yes |
| 19 | NDC80 |  |  | 27 | 0.695 | 0.1651 | Yes |
| 20 | ATAD2 |  |  | 28 | 0.685 | 0.1731 | Yes |
| 21 | KIF15 |  |  | 29 | 0.684 | 0.1811 | Yes |
| 22 | ARHGAP11A |  |  | 30 | 0.683 | 0.1891 | Yes |
| 23 | PCLAF |  |  | 31 | 0.681 | 0.1971 | Yes |
| 24 | SGO1 |  |  | 32 | 0.677 | 0.2050 | Yes |
| 25 | KPNA2 |  |  | 33 | 0.676 | 0.2129 | Yes |
| 26 | CENPF |  |  | 34 | 0.670 | 0.2207 | Yes |
| 27 | KNL1 |  |  | 35 | 0.666 | 0.2285 | Yes |
| 28 | SGO2 |  |  | 36 | 0.664 | 0.2362 | Yes |
| 29 | KIF4A |  |  | 37 | 0.662 | 0.2440 | Yes |
| 30 | ANLN |  |  | 39 | 0.659 | 0.2500 | Yes |
| 31 | RAD51AP1 |  |  | 40 | 0.657 | 0.2576 | Yes |
| 32 | SMC4 |  |  | 41 | 0.655 | 0.2653 | Yes |
| 33 | MELK |  |  | 42 | 0.655 | 0.2729 | Yes |
| 34 | CENPE |  |  | 43 | 0.654 | 0.2806 | Yes |
| 35 | NUF2 |  |  | 44 | 0.651 | 0.2882 | Yes |
| 36 | PRR11 |  |  | 45 | 0.649 | 0.2958 | Yes |
| 37 | DLGAP5 |  |  | 46 | 0.647 | 0.3033 | Yes |
| 38 | KIFC1 |  |  | 47 | 0.645 | 0.3109 | Yes |
| 39 | DHFR |  |  | 49 | 0.638 | 0.3166 | Yes |
| 40 | CENPA |  |  | 51 | 0.636 | 0.3224 | Yes |
| 41 | CDCA5 |  |  | 52 | 0.632 | 0.3297 | Yes |
| 42 | CKAP2 |  |  | 53 | 0.628 | 0.3371 | Yes |
| 43 | CCNA2 |  |  | 54 | 0.626 | 0.3444 | Yes |
| 44 | AURKA |  |  | 55 | 0.617 | 0.3516 | Yes |
| 45 | FANCI |  |  | 56 | 0.615 | 0.3588 | Yes |
| 46 | MYBL2 |  |  | 58 | 0.612 | 0.3643 | Yes |
| 47 | CKAP2L |  |  | 59 | 0.611 | 0.3714 | Yes |
| 48 | CENPU |  |  | 60 | 0.605 | 0.3785 | Yes |
| 49 | CENPK |  |  | 62 | 0.603 | 0.3838 | Yes |
| 50 | TYMS |  |  | 63 | 0.601 | 0.3908 | Yes |
| 51 | BUB1B |  |  | 64 | 0.596 | 0.3978 | Yes |
| 52 | CCDC34 |  |  | 65 | 0.595 | 0.4047 | Yes |
| 53 | KIF2C |  |  | 66 | 0.591 | 0.4116 | Yes |
| 54 | SMC2 |  |  | 68 | 0.586 | 0.4168 | Yes |
| 55 | CDK1 |  |  | 69 | 0.585 | 0.4236 | Yes |
| 56 | H2AFX |  |  | 71 | 0.576 | 0.4287 | Yes |
| 57 | CKS1B |  |  | 72 | 0.575 | 0.4354 | Yes |
| 58 | CIP2A |  |  | 73 | 0.574 | 0.4421 | Yes |
| 59 | RACGAP1 |  |  | 75 | 0.572 | 0.4471 | Yes |
| 60 | FOXM1 |  |  | 76 | 0.570 | 0.4537 | Yes |
| 61 | MAD2L1 |  |  | 77 | 0.569 | 0.4604 | Yes |
| 62 | PLK1 |  |  | 78 | 0.567 | 0.4670 | Yes |
| 63 | DBF4 |  |  | 79 | 0.567 | 0.4736 | Yes |
| 64 | CDCA8 |  |  | 82 | 0.562 | 0.4768 | Yes |
| 65 | CKS2 |  |  | 83 | 0.558 | 0.4834 | Yes |
| 66 | ECT2 |  |  | 84 | 0.558 | 0.4899 | Yes |
| 67 | KIF14 |  |  | 85 | 0.557 | 0.4964 | Yes |
| 68 | UBE2S |  |  | 87 | 0.555 | 0.5012 | Yes |
| 69 | AURKB |  |  | 89 | 0.548 | 0.5059 | Yes |
| 70 | SPC24 |  |  | 91 | 0.544 | 0.5106 | Yes |
| 71 | CENPM |  |  | 92 | 0.544 | 0.5169 | Yes |
| 72 | CCNB1 |  |  | 93 | 0.544 | 0.5233 | Yes |
| 73 | NCAPH |  |  | 94 | 0.543 | 0.5296 | Yes |
| 74 | PBK |  |  | 95 | 0.540 | 0.5359 | Yes |
| 75 | DEPDC1 |  |  | 97 | 0.536 | 0.5405 | Yes |
| 76 | SKA3 |  |  | 99 | 0.525 | 0.5450 | Yes |
| 77 | RRM1 |  |  | 103 | 0.520 | 0.5460 | Yes |
| 78 | MXD3 |  |  | 104 | 0.519 | 0.5520 | Yes |
| 79 | CDKN3 |  |  | 108 | 0.513 | 0.5530 | Yes |
| 80 | UBE2T |  |  | 110 | 0.512 | 0.5573 | Yes |
| 81 | CDCA3 |  |  | 111 | 0.511 | 0.5632 | Yes |
| 82 | DIAPH3 |  |  | 112 | 0.510 | 0.5692 | Yes |
| 83 | BUB1 |  |  | 113 | 0.508 | 0.5751 | Yes |
| 84 | TUBB4B |  |  | 114 | 0.507 | 0.5811 | Yes |
| 85 | RAD21 |  |  | 115 | 0.506 | 0.5870 | Yes |
| 86 | BIRC5 |  |  | 116 | 0.504 | 0.5929 | Yes |
| 87 | CLSPN |  |  | 117 | 0.503 | 0.5987 | Yes |
| 88 | DTYMK |  |  | 118 | 0.503 | 0.6046 | Yes |
| 89 | ASF1B |  |  | 120 | 0.501 | 0.6088 | Yes |
| 90 | PSMC3IP |  |  | 122 | 0.495 | 0.6129 | Yes |
| 91 | SPDL1 |  |  | 126 | 0.492 | 0.6136 | Yes |
| 92 | TMPO |  |  | 127 | 0.492 | 0.6193 | Yes |
| 93 | ITGB3BP |  |  | 128 | 0.490 | 0.6251 | Yes |
| 94 | LMNB2 |  |  | 129 | 0.488 | 0.6308 | Yes |
| 95 | SHCBP1 |  |  | 133 | 0.484 | 0.6314 | Yes |
| 96 | GMNN |  |  | 135 | 0.484 | 0.6353 | Yes |
| 97 | NCAPD2 |  |  | 136 | 0.483 | 0.6410 | Yes |
| 98 | TTK |  |  | 137 | 0.482 | 0.6466 | Yes |
| 99 | C21orf58 |  |  | 142 | 0.470 | 0.6454 | Yes |
| 100 | ORC6 |  |  | 150 | 0.461 | 0.6390 | Yes |
| 101 | KIF22 |  |  | 152 | 0.459 | 0.6427 | Yes |
| 102 | NCAPD3 |  |  | 153 | 0.458 | 0.6480 | Yes |
| 103 | KNSTRN |  |  | 155 | 0.456 | 0.6517 | Yes |
| 104 | SPAG5 |  |  | 156 | 0.455 | 0.6570 | Yes |
| 105 | CENPQ |  |  | 163 | 0.446 | 0.6521 | Yes |
| 106 | CDKN2C |  |  | 164 | 0.445 | 0.6573 | Yes |
| 107 | TK1 |  |  | 167 | 0.444 | 0.6591 | Yes |
| 108 | PIMREG |  |  | 169 | 0.443 | 0.6626 | Yes |
| 109 | ATAD5 |  |  | 170 | 0.442 | 0.6678 | Yes |
| 110 | BRCA1 |  |  | 171 | 0.440 | 0.6729 | Yes |
| 111 | CDK5RAP2 |  |  | 173 | 0.438 | 0.6763 | Yes |
| 112 | MKI67 |  |  | 179 | 0.436 | 0.6730 | Yes |
| 113 | FEN1 |  |  | 180 | 0.435 | 0.6781 | Yes |
| 114 | CDC20 |  |  | 184 | 0.431 | 0.6781 | Yes |
| 115 | USP1 |  |  | 185 | 0.430 | 0.6831 | Yes |
| 116 | MIS18BP1 |  |  | 186 | 0.430 | 0.6881 | Yes |
| 117 | CDKN2D |  |  | 190 | 0.427 | 0.6881 | Yes |
| 118 | HMMR |  |  | 191 | 0.426 | 0.6931 | Yes |
| 119 | CKAP5 |  |  | 192 | 0.426 | 0.6980 | Yes |
| 120 | MND1 |  |  | 193 | 0.424 | 0.7030 | Yes |
| 121 | NEK2 |  |  | 194 | 0.423 | 0.7079 | Yes |
| 122 | CCP110 |  |  | 204 | 0.411 | 0.6976 | No |
| 123 | ZWINT |  |  | 212 | 0.407 | 0.6906 | No |
| 124 | MNS1 |  |  | 219 | 0.401 | 0.6852 | No |
| 125 | POC1A |  |  | 234 | 0.390 | 0.6662 | No |
| 126 | EZH2 |  |  | 236 | 0.388 | 0.6691 | No |
| 127 | PARPBP |  |  | 240 | 0.387 | 0.6685 | No |
| 128 | TRIP13 |  |  | 241 | 0.387 | 0.6731 | No |
| 129 | RTKN2 |  |  | 248 | 0.379 | 0.6674 | No |
| 130 | CCNB2 |  |  | 250 | 0.379 | 0.6702 | No |
| 131 | CENPW |  |  | 267 | 0.366 | 0.6475 | No |
| 132 | TUBA1C |  |  | 270 | 0.362 | 0.6484 | No |
| 133 | ANP32E |  |  | 280 | 0.358 | 0.6375 | No |
| 134 | CDC25B |  |  | 286 | 0.356 | 0.6332 | No |
| 135 | ZWILCH |  |  | 292 | 0.353 | 0.6289 | No |
| 136 | NSD2 |  |  | 303 | 0.346 | 0.6162 | No |
| 137 | CENPN |  |  | 309 | 0.343 | 0.6118 | No |
| 138 | OIP5 |  |  | 315 | 0.341 | 0.6073 | No |
| 139 | PHF19 |  |  | 319 | 0.339 | 0.6063 | No |
| 140 | RFC3 |  |  | 322 | 0.338 | 0.6068 | No |
| 141 | HIRIP3 |  |  | 336 | 0.331 | 0.5889 | No |
| 142 | BTG3 |  |  | 337 | 0.331 | 0.5927 | No |
| 143 | LMNB1 |  |  | 363 | 0.321 | 0.5545 | No |
| 144 | XPO1 |  |  | 365 | 0.321 | 0.5565 | No |
| 145 | UACA |  |  | 368 | 0.320 | 0.5569 | No |
| 146 | SOGA1 |  |  | 378 | 0.314 | 0.5454 | No |
| 147 | KIF20A |  |  | 392 | 0.311 | 0.5272 | No |
| 148 | TEDC1 |  |  | 399 | 0.309 | 0.5207 | No |
| 149 | RHEB |  |  | 404 | 0.307 | 0.5176 | No |
| 150 | PTTG1 |  |  | 413 | 0.302 | 0.5077 | No |
| 151 | PHIP |  |  | 440 | 0.295 | 0.4674 | No |
| 152 | G2E3 |  |  | 446 | 0.294 | 0.4624 | No |
| 153 | CBX5 |  |  | 457 | 0.291 | 0.4490 | No |
| 154 | APOLD1 |  |  | 487 | 0.284 | 0.4036 | No |
| 155 | CENPH |  |  | 501 | 0.281 | 0.3850 | No |
| 156 | FAM83D |  |  | 511 | 0.279 | 0.3732 | No |
| 157 | CDC27 |  |  | 520 | 0.278 | 0.3630 | No |
| 158 | SAC3D1 |  |  | 529 | 0.276 | 0.3528 | No |
| 159 | NUCB2 |  |  | 533 | 0.275 | 0.3509 | No |
| 160 | POLR2A |  |  | 545 | 0.273 | 0.3356 | No |
| 161 | ALYREF |  |  | 546 | 0.273 | 0.3388 | No |
| 162 | SAP30 |  |  | 547 | 0.273 | 0.3420 | No |
| 163 | GGH |  |  | 564 | 0.270 | 0.3183 | No |
| 164 | RPA3 |  |  | 584 | 0.267 | 0.2894 | No |
| 165 | EXOSC8 |  |  | 647 | 0.256 | 0.1882 | No |
Table: GSEA details [plain text format]

  

Fig 2: DOROSZKO\_U3013MG\_CLUSTER\_2: Random ES distribution      
 Gene set null distribution of ES for **DOROSZKO\_U3013MG\_CLUSTER\_2**
